# Living in immigrant communities does not impact total knee arthroplasty outcomes: experience from a high-volume center in the United States

**DOI:** 10.1186/s12891-019-2446-y

**Published:** 2019-02-09

**Authors:** Bella Mehta, Jackie Szymonifka, Shirin Dey, Iris Navarro-Millan, Stephen Grassia, Lisa A. Mandl, Anne R. Bass, Linda Russell, Michael Parks, Mark Figgie, Lily Lee, Joe Nguyen, Susan M. Goodman

**Affiliations:** 1 Hospital for Special Surgery, 535 E 70th Street, New York, NY 10021 USA; 2 Weill Cornell Medicine, New York, USA

## Abstract

**Background:**

Community characteristics such as poverty affect total knee arthroplasty (TKA) outcomes. However, it is unknown whether other community factors such as immigrant proportion (IP) also affect outcomes. Our objective was to determine the association of neighborhood IP on preoperative (pre-op) and 2-year postoperative (post-op) Western Ontario and McMaster Universities Osteoarthritis Index (WOMAC) pain and function after elective TKA.

**Methods:**

Patients in a high volume institutional TKA registry between May 2007 and February 2011 were retrospectively analyzed. Demographics, pre-op and 2-year post-op WOMAC pain and function scores, and geocodable addresses were obtained. Patient-level variables were linked to US Census Bureau census tract data. The effect of patient and neighborhood-level factors on WOMAC scores were analyzed using linear mixed effects models.

**Results:**

3898 TKA patients were analyzed. Pre-op and 2-year post-op WOMAC pain and function scores were between 2.75–4.88 WOMAC points worse in neighborhoods with a high IP (≥ 40%) compared to low IP (< 10%). In multivariable analyses, these differences were not statistically significant. Women had worse pre-op and 2-year post-op WOMAC scores (all *p* ≤ 0.04), but this difference was not influenced by neighborhood IP (all p_interaction_ NS).

**Conclusions:**

Patients living in high (≥40%) IP neighborhoods do not have worse pre-op or 2-year post-op pain and function outcomes after TKA compared to those living in low (< 10%) IP neighborhoods. Although sex differences favoring males are notable, these differences are not associated with IP. High neighborhood IP do not appear to affect outcomes after TKA.

## Background

Social factors at the individual and community level affect health, including total knee arthroplasty (TKA) outcomes in osteoarthritis (OA) [1, 2]. The definitive therapy for advanced symptomatic knee OA is TKA, yet 30% of patients report persistent pain or dissatisfaction with their outcomes [3–7]. Research exploring poor outcomes after TKA have identified social risk factors at the individual level such as race, gender, or education, and community characteristics such as race and poverty [1, 2, 8]. Immigrants are increasingly recognized to be among these vulnerable groups in multiple medical arenas, for example, immigrants have higher perinatal mortality. They have a lower breast cancer, colon cancer, ophthalmology and visual acuity screening rates with differences in preventive care between immigrants and US natives [9–11]. At the community level, those from communities with high immigrant proportions present with more advanced colon cancer leading to poorer surgical outcomes, and cardiovascular disease risk is increased in some immigrant communities [12, 13]. Moreover, obtaining accurate information on immigrant status at the individual level can be fraught for the individual who may not have complete documentation of their immigration status, so community level data on immigrant proportion may be useful to study.

By 2023 the immigrant population will account for more than one in seven U.S. residents (51 million), and they currently comprise 17% of the US labor force, underscoring the importance of understanding the factors influencing the health status of immigrants [14, 15]. Immigrants are more likely to reside in communities with a high proportion of immigrants. New York City has a large immigrant population with multiple immigrant neighborhoods, and New York State has the highest overall proportion of immigrants, providing an ideal locale to study the impacts of immigrant communities on health outcomes [16]. Given the rising utilization of TKA, social factors that may impact outcomes are significant at the individual, community, and public health level [17–19].

Census tracts are small geographical areas containing approximately 4000 individuals, that are designed to be homogeneous, and therefore can be used to analyze neighborhood level data [20]. The American Community Survey/census bureau regularly collects data on multiple factors including poverty, race, education, and immigrant status. This permits use of data from each census tract to estimate the proportion of immigrants in a neighborhood and to study health outcomes in relation to the immigrant proportion, or other social factors [13, 21]. Using geospatial localization, individual level data can be linked to specific census tracts, so we can analyze individual health outcomes across a gradient determined by the community factors, comparing outcomes in census tracts where the proportion of immigrants is high vs. low. Using geospatial localization to link individual level data to specific census tracts, we can analyze individual health outcomes across a gradient of community factors including the proportion of immigrants in a census tract as determined by the American Community Survey. It is unknown whether the proportion of the immigrants in a neighborhood influence outcome in TKA. Our objective was to study the association between neighborhood immigrant proportion (IP) and, pain and function outcomes after TKA.

## Methods

### Study design

This is a retrospective study which was conducted using prospectively acquired longitudinal registry data from a large volume orthopedic hospital. The hospital mainly serves the tristate area (New York, New Jersey and Connecticut), which has a high density of immigrants, permitting us to study the association of neighborhood immigrant proportions on TKA outcomes [22].

### Registry/data

Patients at least 18 years old were prospectively enrolled in the registry between May 1, 2007 and July 1, 2011 after providing informed consent. Patients included in this study completed questionnaires, including patient reported outcomes, both prior to TKA and 2 years after TKA. Registry patients who lived outside the tristate area were excluded from our analysis so as to focus on the main catchment area for the hospital and permit greater generalizability by excluding patients with resources to travel long distances for care. We excluded patients who underwent revision TKA, bilateral TKA and patients who had a contralateral TKA within 2 years of the index surgery so that the study reflects outcomes of the index surgery. We also excluded patients who had TKA secondary to fractures, avascular necrosis or inflammatory diseases such as rheumatoid arthritis, psoriatic arthritis and ankylosing spondylitis for the study to represent OA patients.

Baseline covariates collected in the registry included date of birth, sex, body mass index (BMI), race (black, white, Asian, other), ethnicity (Hispanic, non-Hispanic), highest education level completed (some high school, high school graduate, some college, college graduate, master’s degree or higher) and living status (alone or with other(s)). The patient-reported outcomes collected in the registry were the pre-operative (pre-op) and 2-year postoperative (post-op) Knee injury and Osteoarthritis Outcome Score (KOOS), from which Western Ontario and McMaster Universities Osteoarthritis Index (WOMAC) pain and function scores are derived, and the Hospital for Special Surgery (HSS) Expectations Survey before surgery. The WOMAC is a validated lower extremity specific scale and commonly used as a patient-reported instrument after TKA. WOMAC results are scored between 0 and 100 points, with higher scores indicating better outcomes. Lower extremity pain and function are assessed using 2 subscales; a higher score indicates better status and a score less than 40 indicates significant pain and poor function [23]. The primary outcomes of the study were 2-year WOMAC pain and function scores. The Hospital for Special Surgery (HSS) Expectations Survey is a validated instrument which is specific for TKA outcomes and assesses multiple domains relevant to TKA [24]. Administrative data including comorbidities and patients’ addresses were collected from hospital records. The addresses were used to permit geocoding to link individual level data to census tract defined area-based measures (ABMs).

### Census tracts/geocoding

**Fig. 1:**
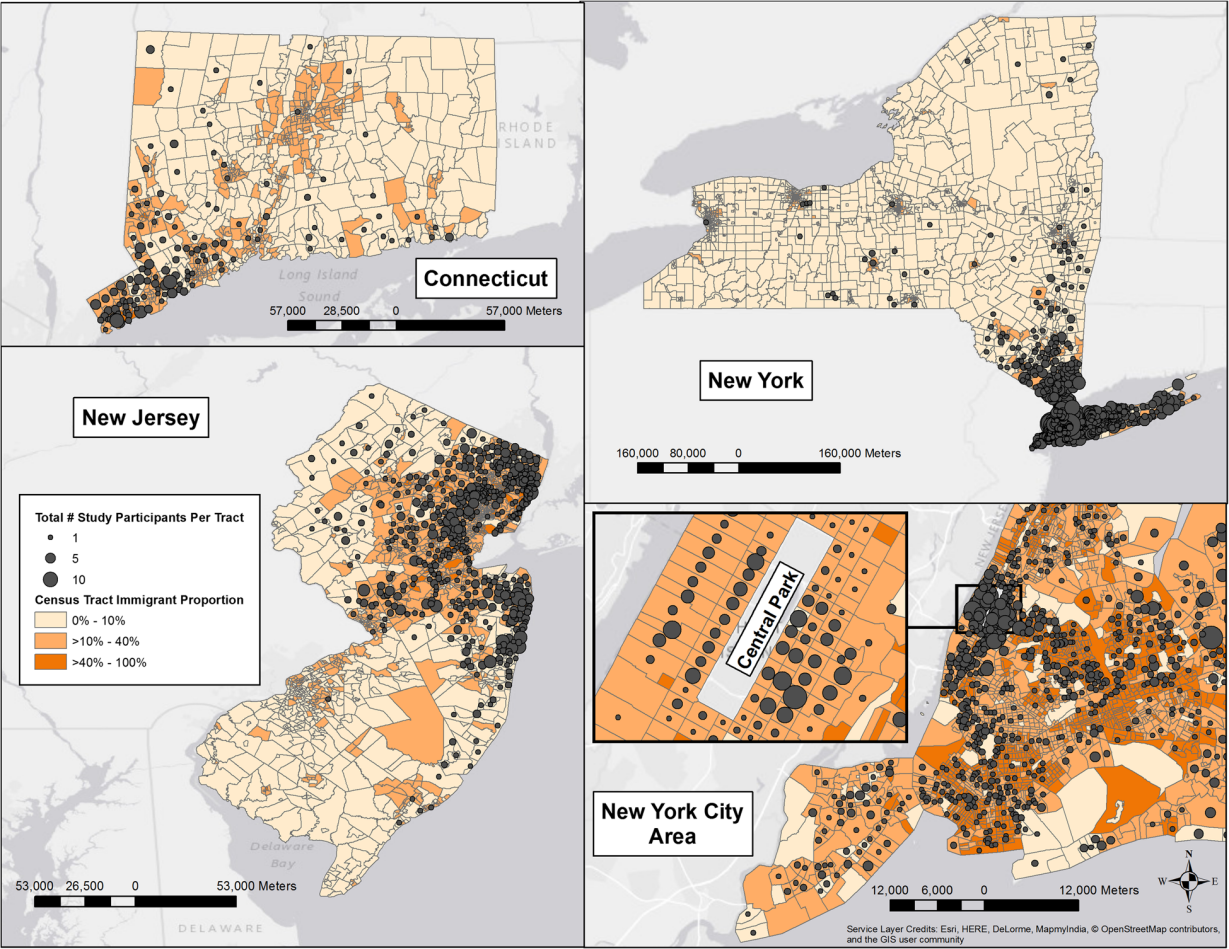
Geospatial localization of study patients who underwent Total Knee Arthroplasty by census tracts in the catchment area of the hospital (Created using ArcGIS software using base maps downloaded from the open access census website: https://www.census.gov/geo/maps-data/data/tiger-line.html)

Geocoding is the process of converting addresses to a precise geographic coordinate (defined by latitude and longitude) which one can use to place markers or positions on a map (Fig. 1). This facilitates spatial analysis using geographic information systems (GIS) and Enterprise Location Intelligence systems. The American Community Survey/Census Bureau release multiple complex datasets containing area-based measures (ABMs) every 10 years. These ABMs are objective data collected by the Census Bureau and broken down into smaller subsections to characterize the neighborhood. This data is presented using smaller subunits - zip codes (which average 150,000 residents), census tracts (average 4000 residents), and census blocks (average 1000 residents). For measuring neighborhood social factors, census tracts are more consistent than census blocks or zip codes [25]. Census tracts are designed to be homogenous with respect to population characteristics, economic status, and living conditions [20].

Each patient in the registry with a geocodable address was assigned to a census tract using ArcGIS [26]. ABMs were then extracted for each census tract using data released by the Census Bureau. Our primary ABM of interest was “foreign born”. The term “foreign-born” and “immigrant” are used interchangeably and refer to anyone who is not a US citizen at birth. This includes naturalized US citizens, lawful permanent residents, temporary migrants, humanitarian migrants, and persons illegally present in the United States [15]. Within each census tract, we categorized the immigrant proportion (IP) as low (< 10%), moderate (10–40%) and high (≥40%), since this categorization has been utilized in similar studies and has been associated with health outcomes [21]. For sensitivity analyses, we dichotomized IP values as < 20% IP and > 20% IP and also kept IP as a continuous variable and performed all primary outcomes analyses. The other ABM included in this study was the percent of the population living below poverty as a measure of neighborhood socioeconomics. The tristate area has a total of 7762 census tracts. (Fig. 1).

### Statistical analysis

Continuous variables are summarized as mean ± standard deviation and compared using t-tests for binary comparisons or ANOVA tests when comparing more than two subgroups. Categorical variables are summarized as frequency and percent and compared using chi-squared tests. Spearman correlation coefficients were calculated for neighborhood poverty and IP to determine the strength of the relationship between these two crucial ABMs. We used descriptive statistics to compare respondents and non-respondents of the 2-year post-op WOMAC scores. Univariate and multivariable linear mixed effects models, with census tracts treated as random effects, were used to analyze the impact of individual and census tract-level factors on pre-op and 2-year post-op WOMAC pain and function scores. Multivariable models were adjusted for individual-level age, sex, BMI, comorbidities (none versus at least one), and living status (alone versus with other(s)), as well as and neighborhood poverty. 2-year post-op models were additionally adjusted for baseline WOMAC scores. Separate linear mixed effects models were calculated to assess the interactions between IP and sex, as well as IP and individual education level. There was less than 1% missing data and thus we did not impute missing values.

Due to the reduced sample size in the ≥40% IP cohort, we performed a matched analysis to further assess the impact of IP after controlling for age, sex, BMI and number of comorbidities. Patients from high IP neighborhoods were matched to one patient from a low IP neighborhood and to one patient from a moderate IP neighborhood. Matching factors included age (within 5 years), sex (perfect match required), BMI (within 1 kg/m2) and number of comorbidities (0 or ≥ 1, perfect match required). Univariate and multivariable linear mixed effect models were performed, treating each matched pair as a random effect. Multivariable models were adjusted for individual living status (alone versus with other(s)) and census tract poverty. SAS version 9.4 was used for the analysis.

## Results

This study included 3898 OA patients who underwent TKA at our single high-volume center. These patients represented 1937 (25%) of census tracts in the tristate area and 1551 of these census tracts were represented by two or more patients. Seven patients were excluded from the study as their addresses could not be geocoded. Baseline characteristics are summarized overall and for patients from neighborhoods with a low IP (*n* = 1032, 26.5%), moderate IP (*n* = 2527, 64.8%), and high IP (*n* = 339, 8.7%) (Table 1). A lower proportion of blacks and Hispanics were from neighborhoods with a low IP than neighborhoods with a high IP, while there was a higher proportion of college graduates in neighborhoods with low IP (all *p* < 0.001). Patients with at least one comorbidity and patients living alone were more prevalent in high IP neighborhoods (*p* = 0.003 and *p* < 0.001, respectively). High IP neighborhoods also had higher rates of poverty (*p* < 0.001). Interestingly, expectations of surgery did not differ between the groups. Correlation between neighborhood poverty and IP was moderate (Spearman *ρ* = 0.43). Overall 2 year response rates for WOMAC pain was 97.1% (115 non responders) and WOMAC function was 96.7% (129 non responders). There were no statistically significant differences in terms of IP between those who responded to 2-year post-op WOMAC scores versus those who did not (Table 2).

**Table 1:** Baseline characteristics of the Total Knee Arthroplasty patients and by neighborhood Immigrant proportion

Characteristic	All primary TKA (*N* = 3898)	Immigrant proportion < 10% (*n* = 1032)	Immigrant proportion 10 to < 40% (*n* = 2527)	Immigrant proportion ≥ 40% (*n* = 339)	*p*-value (< 10% v. ≥ 40%)
Patient demographics					
Age at surgery (years), mean ± SD	67.8 ± 9.5	67.0 ± 9.2	68.1 ± 9.6	67.8 ± 9.6	0.16
Sex: female, n (%)	2346 (60.2%)	568 (55.0%)	1538 (60.9%)	240 (70.8%)	< 0.001
BMI (kg/m^2^), mean ± SD	30.0 ± 5.9	30.1 ± 5.7	29.9 ± 6.0	31.0 ± 6.6	0.03
Race, *n* (%)					< 0.001
White	3568 (91.5%)	1012 (98.1%)	2297 (90.9%)	259 (76.4%)	
Black	174 (4.5%)	5 (0.5%)	128 (5.1%)	41 (12.1%)	
Asian	74 (1.9%)	9 (0.9%)	47 (1.9%)	18 (5.3%)	
Other	56 (1.4%)	5 (0.5%)	35 (1.4%)	16 (4.7%)	
Unknown	26 (0.7%)	1 (0.1%)	20 (0.8%)	5 (1.5%)	
Ethnicity, *n* (%)					< 0.001
Hispanic	129 (3.3%)	5 (0.5%)	35 (1.4%)	16 (4.7%)	
Patient status					
One or more comorbidities	1057 (27.1%)	267 (25.9%)	674 (26.7%)	116 (34.2%)	0.003
Sociodemographic characteristics					
Education level (highest), *n* (%)					< 0.001
Some high school, high school graduate or some college (< College graduate)	1366 (35.6%)	359 (36.0%)	849 (35.1%)	158 (49.4%)	
College graduate or Masters, professional or doctorate degree (> College graduate)	2370 (63.4%)	639 (64.0%)	1569 (64.9%)	162 (50.6%)	
Lives alone, *n* (%)	860 (22.4%)	159 (15.6%)	598 (24.1%)	103 (31.0%)	< 0.001
Census tract characteristics					
Number of census tracts, *n* (%)	1937 (100%)	498 (25.7%)	1196 (61.7%)	243 (12.5%)	
Poverty, *n* (%)					< 0.001
< 10%	3175 (81.5%)	975 (94.5%)	2078 (82.2%)	122 (36.0%)	
10% – < 20%	511 (13.1%)	48 (4.7%)	300 (11.9%)	163 (48.1%)	
≥ 20%	212 (5.4%)	9 (0.9%)	149 (5.9%)	54 (15.9%)	
Patient-reported outcomes					
HSS expectation survey, mean ± SD	79.2 ± 17.6	80.1 ± 17.7	78.8 ± 17.4	79.5 ± 18.3	0.63
Pre-operative survey results, mean ± SD					
WOMAC pain	54.4 ± 17.5	54.9 ± 17.6	54.7 ± 17.3	51.0 ± 18.9	< 0.001
WOMAC function	53.7 ± 17.6	54.2 ± 16.9	54.1 ± 17.7	49.1 ± 17.3	< 0.001
2-year post-operative survey results					
WOMAC pain, mean ± SD	87.9 ± 15.6	89.3 ± 14.7	87.5 ± 15.7	86.5 ± 17.5	0.01
WOMAC function, mean ± SD	85.6 ± 16.1	86.7 ± 14.9	85.4 ± 16.2	83.6 ± 17.9	0.005

*TKA* total knee arthroplasty, *SD* standard deviation, *BMI* body mass index, *ASA* American Society of Anesthesiologists, *HSS* Hospital for Special Surgery, *WOMAC* Western Ontario and McMaster Universities Osteoarthritis Index

**Table 2:** Description of non-responders and responders for 2-year WOMAC pain and function outcomes across census tract Immigrant Proportion (IP)

Census tract IP, *n* (%)	Without 2-year WOMAC pain [Non-responder]	With 2-year WOMAC pain [Responder]	*p*-value
< 10%	28.7%	26.4%	0.085
10% – < 40%	57.4%	65.1%	
≥ 40%	13.9%	8.5%	
Census tract IP, *n* (%)	Without 2-year WOMAC function [Non-Responder]	With 2-year WOMAC function [Responder]	*p*-value
< 10%	27.9%	26.4%	0.142
10% – < 40%	58.9%	65.0%	
≥ 40%	13.2%	8.6%	

*WOMAC* Western Ontario and McMaster Universities Osteoarthritis Index, *IP* Immigrant Proportion

In univariate analyses, age, sex, BMI, education levels, and IP were associated with pre-op and 2-year post-op WOMAC pain and function (Table 3). Pre-op and 2-year post-op WOMAC pain and function scores were between 2.75 to 4.88 WOMAC points worse in neighborhoods with a high IP compared to low IP. These differences were small, yet statistically significant (all *p* ≤ 0.006) and there was a stepwise decrease in scores in high IP neighborhoods suggesting a “dose-response” effect.

**Table 3:** Impact of neighborhood immigrant proportion (IP) on WOMAC pain and function (unadjusted/ univariate models)

	Preoperative estimate ± standard error				Postoperative 2-year estimate ± standard error			
	WOMAC pain	*p*-value	WOMAC function	*p*- value	WOMAC pain	*p*-value	WOMAC function	*p*-value
Census Tract immigrant proportion (IP)								
< 10%	reference	–	reference	–	reference	–	reference	–
10% – < 40%	−0.21 ± 0.69	0.76	−0.14 ± 0.70	0.84	−1.79 ± 0.58	0.002	− 1.32 ± 0.60	0.03
≥ 40%	−3.60 ± 1.15	0.002	−4.88 ± 1.15	< 0.001	− 2.75 ± 1.00	0.006	−3.10 ± 1.03	0.003
Age, years	0.38 ± 0.03	< 0.001	0.15 ± 0.03	< 0.001	0.06 ± 0.03	0.02	− 0.32 ± 0.04	< 0.001
BMI, kg/m^2^	− 0.62 ± 0.05	< 0.001	− 0.73 ± 0.05	< 0.001	− 0.24 ± 0.04	< 0.001	− 0.32 ± 0.04	< 0.001
Sex: female	−5.94 ± 0.57	< 0.001	−6.66 ± 0.57	< 0.001	− 2.62 ± 0.52	< 0.001	− 2.65 ± 0.53	< 0.001
Comorbidities								
One or more	−1.15 ± 0.64	0.07	− 2.01 ± 0.64	0.002	−1.91 ± 0.57	< 0.001	− 3.10 ± 0.59	< 0.001
None	reference	–	reference	–	reference	–	reference	–
Census tract poverty								
< 10%	4.24 ± 1.30	0.001	3.80 ± 1.30	0.004	− 3.50 ± 0.73	< 0.001	− 3.36 ± 0.76	< 0.001
10% – < 20%	0.94 ± 1.49	0.53	− 0.49 ± 1.49	0.74	−1.61 ± 0.64	0.01	− 1.75 ± 0.67	0.009
≥ 20%	reference	–	reference	–	reference	–	reference	–
Education level								
< College graduate	−7.57 ± 0.37	< 0.001	−8.82 ± 0.59	< 0.001	− 3.88 ± 0.53	< 0.001	−3.95 ± 0.55	< 0.001
≥ College graduate	reference	–	reference	–	reference	–	reference	–
Live alone								
No	0.63 ± 0.69	0.36	2.57 ± 0.69	< 0.001	2.22 ± 0.61	< 0.001	2.68 ± 0.63	< 0.001
Yes	reference	–	reference	–	reference	–	reference	–

*WOMAC* Western Ontario and McMaster Universities Osteoarthritis Index, *BMI* body mass index

In multivariable analyses adjusted for age, sex, number of comorbidities, neighborhood poverty, individuals living alone, patients from neighborhoods with low IP had showed no difference in WOMAC pain and function scores at either pre-op or 2-years post-op (after adjustment for baseline WOMAC scores) as compared to those from high IP neighborhoods (Table 4). While women and patients with less than college education had worse pre-op and 2-year post-op WOMAC pain and function scores (all *p* ≤ 0.04), this difference was not significantly influenced by IP (all p_interaction_ NS). Thus, individual level factors may explain between IP level differences in TKA outcomes.

**Table 4:** Multivariable models comparing WOMAC pain and function by neighborhood immigrant proportion (IP)

Timepoint	WOMAC pain Mean ± SE	*p*-value	WOMAC function Mean ± SE	*p*-value
Preoperative		0.29		0.10
Immigrant Proportion < 10%	52.56 ± 1.07		51.78 ± 1.06	
Immigrant Proportion ≥ 40%	51.32 ± 1.22		49.88 ± 1.21	
2-year Postoperative		0.60		0.58
Immigrant Proportion < 10%	87.19 ± 0.98		84.49 ± 0.98	
Immigrant Proportion ≥ 40%	86.62 ± 1.13		83.89 ± 1.13	

*SE* standard error, *WOMAC* Western Ontario and McMaster Universities Osteoarthritis Index

Multivariable models adjusted for age, sex, comorbidities (none vs. ≥ 1), live alone, and census tract percent poverty. 2-year models included additional adjustment for BMI and corresponding baseline WOMAC pain or function

We performed sensitivity analyses using different thresholds for IP, comparing < 20% versus > 20%, and also treating IP as a continuous variable. Results were similar to the primary endpoint results described above. We performed an additional sensitivity analysis since only 9% of our study sample was from high IP neighborhoods. A 1:1:1 match of patients from neighborhoods with a high, moderate and low IP was performed. We analyzed 289 matched sets comprised of 867 patients. We performed linear mixed effects modeling in a similar manner to the primary analyses. In univariate analyses, both pre-op and 2-year post-op WOMAC pain and function scores were 1–6 points worse in the neighborhoods with a high IP compared to low IP (Table 5). After adjusting for individual education level and living status (alone or with other(s)), as well as neighborhood poverty, multivariable modeling revealed nonsignificant differences, similar to our primary analyses.

**Table 5:** Sensitivity analysis comparing WOMAC pain and function by Immigrant proportion subgroups after matching

	WOMAC pain Estimate ± SE	*p*-value	WOMAC function Estimate ± SE	*p*-value
Pre-operative, univariate				
Immigrant proportion				
< 10%	reference	–	reference	–
10% – < 40%	−1.34 ± 1.47	0.360	−2.42 ± 1.41	0.087
≥ 40%	−4.21 ± 1.47	0.004	−5.98 ± 1.42	< 0.001
Pre-operative, multivariable^a^				
Immigrant proportion				
< 10%	reference	–	reference	–
10% – < 40%	−0.66 ± 1.50	0.659	− 1.67 ± 1.44	0.244
≥ 40%	−1.47 ± 1.76	0.403	− 2.76 ± 1.67	0.098
2-year post-operative, univariate				
Immigrant proportion				
< 10%	reference	–	reference	–
10% – < 40%	0.03 ± 1.26	0.978	−1.05 ± 1.33	0.429
≥ 40%	−1.97 ± 1.26	0.119	− 2.25 ± 1.34	0.094
2-year post-operative, multivariable^a^				
Immigrant proportion				
< 10%	reference	–	Reference	–
10% – < 40%	0.37 ± 1.29	0.772	− 0.60 ± 1.37	0.659
≥ 40%	−1.58 ± 1.52	0.298	− 1.13 ± 1.60	0.482

*SE* standard error, *WOMAC* Western Ontario and McMaster Universities Osteoarthritis Index

Patients were matched on age, sex, BMI and comorbidities (none v. ≥ 1)

^a^Multivariable models adjusted for education level, live alone and census tract percent poverty

## Discussion

Worse health outcomes among immigrants have been described in surgical outcomes in colon cancer and in neonatal health [21, 27], but literature on arthroplasty outcomes in relation to immigrant status is sparse. Outcomes after arthroplasty were assessed in a Swedish cohort, and immigrants reported more pain on the visual analog scale and scored worse in all generic quality of life dimensions compared to non-immigrants [10, 28]. To our knowledge, only one study conducted in the United States addressed disparities among immigrants in use of total joint arthroplasty. In that study, Chinese patients had 22.6 times the odds of declining physician recommended arthroplasty compared to Caucasian patients, after controlling for age, gender, education and socioeconomic status, but no studies have evaluated arthroplasty outcomes among immigrants to the US [29].

It is possible that poor outcomes for immigrants may be associated with other factors associated with poor health outcomes, such as poverty or poor English language proficiency that is also seen in communities with high immigrant proportions. We therefore speculated that immigrant status, captured by the census tract, might similarly increase the risk of poor outcomes among TKA patients [1, 30]. Our study showed that patients coming from neighborhoods with a high proportion of immigrants do not have worse pre-operative or post-operative WOMAC pain and function comparted to patients from neighborhoods with lower immigrant proportions. Thus, individual factors rather than neighborhood immigrant proportion may play a larger role in affecting TKA outcomes. Poor pre-operative status has been reported for patients from other high risk communities prior to TKA, suggesting delay in presentation until their condition is more advanced, but this was not seen in patients from communities with a high IP [1, 31]. Equivalent pain and function outcomes were seen despite increased poverty and less education among those in communities with higher proportion of immigrants.

There are several possible explanations for this finding. In high volume centers such as ours, social factors contributing to health outcomes may be less important than in lower volume centers where the majority of TKA are performed [32].

Our study is limited by the small sample size (*n* = 339) in neighborhoods with a high IP (≥ 40%). However, our primary results were similar in a matched sensitivity analysis, decreasing the risk of bias due to small numbers in the high IP cohort. Another limitation is that this is a single center study from a high-volume hospital, and thus may not be generalizable. While this does eliminate confounding introduced by low surgeon or hospital volume we were unable to evaluate a potential interaction between IP and hospital’s volume status [33, 34]. As only patients with 2-year follow-up data were included in the analysis, there is potential selection bias, which could underestimate the number with poorer outcomes as patients with worse outcomes are less likely to return questionnaires. Further selection bias could have been introduced via differential response rates across IP areas. However, no significant differences were observed when comparing WOMAC responders and non-responders across IP areas at 2-year follow up. An additional limitation is our lack of individual immigration status, which is not routinely collected in hospitals or in registries, although could potentially affect outcomes. However individual birth location would not necessarily correlate the resources available, perceptions about surgery, barriers to care or optimal surgical outcomes which may exist in immigrant communities. Finally, other community factors known to impact health outcomes such as poverty may also play a role in immigrant communities.

This study had a number of strengths. The tristate area has a high concentration of immigrants, particularly New York has the highest proportion of immigrants among all states. While our hospital population is relatively homogenous, the New York environment is not, affording us a unique opportunity to assess the impact of IP on TKA outcomes. We used a well validated and robust TKA register and used both individual and community data to perform this study.

In conclusion, we found no difference in TKA outcomes between those living in communities with higher or lower proportions of immigrants. Multicenter studies with a range of high and low volume TKA centers and more individual level data on immigrant status would be required to further understand the impact of immigrant proportions on TKA outcomes. Prospective studies which approach and evaluate health care disparities from different perspectives are needed to achieve health equity.
